# An In Vitro Study of Fluid Contaminations Influences on Reverse Torque Values of Implant-Abutment Connections

**DOI:** 10.1155/2022/4111710

**Published:** 2022-03-08

**Authors:** Shuying Yang, Yuan Qin, Xueyan Guo, Yaqi Li

**Affiliations:** ^1^State Key Laboratory of Military Stomatology & National Clinical Research Center for Oral Diseases & Shaanxi International Joint Research Center for Oral Diseases, Fourth Military Medical University, Xi'an, 710032, China; ^2^Department of Stomatology, Xi'an Children's Hospital, Affiliated Children's Hospital, Xi'an Jiaotong University, 710003, China

## Abstract

**Purpose:**

To examine the effects of fluid contamination on the reverse torque value (RTV) of abutment screws. 484 titanium fixtures were mounted into the stainless-steel holders.

**Methods:**

11 groups (44 specimens in each group) of implants were mounted in acrylic resin. Ten groups of fixture screw holes were contaminated with chlorhexidine, saliva, blood, fluoride, or combination groups, and one group served as a control without contamination. To simulate the oral environment, samples were subjected to thermal cycling and cyclic loading.

**Results:**

The RTV means were less than the initial torque in both control and contamination groups. The maximum RTV mean was observed in the fluoride group (26.00 ± 1.02 Ncm). In other groups, this rate for control, blood, saliva, and chlorhexidine groups were 18.00 ± 1.78 Ncm, 22.12 ± 1.56 Ncm, 21.56 ± 1.43 Ncm, and 21.89 ± 1.02 Ncm, respectively. In combination groups, the maximum RTV mean was observed in the saliva+CHX group (23.89 ± 1.92 Ncm). In other combination groups, this rate for the blood+CHX, blood+saliva, saliva+fluoride, fluoride+CHX, and fluoride+blood groups were 22.56 ± 1.73 Ncm, 22.00 ± 1.54 Ncm, 20.11 ± 1.58 Ncm, 23.51 ± 1.19 Ncm, 21.02 ± 1.38 Ncm, and 20.11 ± 1.58 Ncm, respectively. The RTV was statistically significant (*p* < 0.05) for the contamination groups (except saliva) and combination groups compared to the control group. There is no statistically significant difference (*p* > 0.05) between the reverse torque value mean of the blood and saliva groups and between that of the fluoride and chlorhexidine groups.

**Conclusion:**

Implant-abutment specimens are suggested to be placed in a saliva environment and should be subjected to cyclic loading.

## 1. Introduction

Studies indicate a remarkable improvement in the life quality of patients who have received dental implants [1]. Dental implants, at present, have a success rate of more than 90%, and this achievement is due to the developments in implant physical design and techniques and improved clinical experience [2]. Advances in implant materials and manufacturing processes cannot reduce the mechanical complications to zero [2]. These mechanical complications can influence dental implant success and include opposing prosthesis fracture, material and component fracture, and screw loosening [3]. Screw loosening has been found to be the most common type of mechanical complication [3–5], ranging from 2% to 45% of abutments [3]. The problem generally occurs during the first year of implant function and is more often reported in the prostheses supported by 2 implants [4]. The screw becomes loose when the clamping force (a force produced by elastic recovery of the dental implant, which pushes the abutment and the dental implant together) is lower than the joint separating force (two major forces to determine screw tightness) [6, 7]. When a screw is tightened by the application of torque (a force applied to a tooth to produce or maintain a crown), it elongates and creates tension [8]. This tension is called preloading, a direct determinant of clamping force [9]. Several factors may affect preload, including screw design and materials, applied torque magnitude [10, 11], torque delivery system [12], and environmental factors affecting the interactions such as presence and type of fluid contaminations and lubrication [13].

Screw loosening can cause different clinical conditions, including the formation of microgap between implant components, displacement of the prosthesis, decreased prosthetic function, fistulae formation, soft tissue infection, and inflammation [14–17]. Considering these potential clinical conditions, screw loosening may be one of the important reasons for an increase in the number of referrals for implant maintenance, which is extremely time-consuming and causes inconvenient problems for the patient [5]. The implant screw hole may become contaminated by blood, saliva, fluoride, and chlorhexidine during clinical and experimental procedures [18]. Over the past years, there have been numerous studies evaluating the behavior of abutment screws after loading. However, few studies have considered the role of fluid contamination on reverse torque (reverse force applied to a tooth to produce root movement) values (RTV) of the abutment screw. Therefore, this study is aimed at examining the effects of blood, saliva, fluoride, and chlorhexidine contaminations, as well as their combined effect on the RTV of abutment screws. Since these fluids are the main probable contaminations in oral conditions, we have chosen them in our study. The null hypothesis of our study was that there is no statistically significant difference in RTV among the contamination groups and the control group. The obtained results provide clinicians with new insight into the effect of probable fluid contaminations in the implant-abutment interface.

## 2. Material and Methods

The current study is experimental research carried out in vitro. 484 titanium fixtures (Grade 4 Titanium, Implantium, Dentium Co., Seoul, Korea: 2021) with 4.3 mm diameter and 10 mm length were mounted into the stainless steel holders (according to the condition based on ISO 14801). The implant-abutment connection type was conical with an internal hexagon. Specimens were divided into 11 groups (*n* = 44). The first test group (A) was not contaminated and used as the control group. In test group B, the implant fixture was contaminated with a capillary blood sample (obtained from a donor by a lancet); in test group C, the implant fixture was contaminated with fresh saliva (it was collected from the same donor at rest); in test group D, the implant fixture was contaminated with chlorhexidine (CHX) (liquid; concentration: 0.2%, pH: 7) (Xi'an Lijun Pharmaceutical Co., Xi'an, China.); and in test group E, implant fixture was contaminated with fluoride (gel, concentration: 0.2%, pH: 6.8) (Xi'an Lijun Pharmaceutical Co., Xi'an, China.). Combinations of contaminations were surveyed in other groups. Contaminants were applied by a pipette until the screw access hole of the implant fixture was completely contaminated. The mean RTV in each group was calculated. Intergroup comparisons were made and calculated for each group. The research was approved based on the Ethics Committee of Xi'an Children's Hospital, Affiliated Children's Hospital, Xi'an Jiaotong University (Approved No. 589766400B), and the participants filled a written informed consent regarding the use of saliva and blood samples.

### 2.1. Reverse Torque Measurement

**Figure 1:**
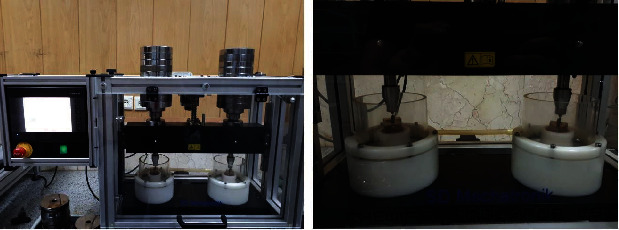
The cyclic loading device (SD Mechatronic, Feld-krichen, Westerham, Germany).

Mounted implants were stabilized by Jig Mounting (a customized rigid metal which used to firmly fix the acrylic sample by tightening metal screws). Initial torque (25 Ncm) was applied with a hand-held torque wrench by one practitioner controlled with a digital torque meter. Each abutment (4.5 mm diameter with 1.5 mm gingival height; Implantium, Dentium Co., Seoul, Korea) was initially tightened with a torque meter (TQ-8800, Lutron Electronic Enterprise Co., Taipei, Taiwan) to 30 Ncm (as recommended by the manufacturer); the second torque value was applied 10 minutes after the initial torque value. To minimize the settling effect, the proper torque was applied and monitored by a digital torque gauge until it reached a value of 30 Ncm. After abutment connection and contamination, implant-abutment assemblies were subjected to thermal cycling: 5°C–55°C, 5000 cycles, and 60 seconds dwell time. To protect the abutments from strain during cyclic loading, we applied abutment covered by a metal base from nickel-chromium alloy (Damcast NB, YADENT Co., China). Samples subjected to cyclic loading and 500000 cycles (100 N cycle and 1 Hertz) were applied to each sample (Figure 1).

### 2.2. Statistical Analysis

RTV values were reported as mean ± SD. We used paired *t*-test to compare values before and after contamination. The one-way ANOVA and Tukey's *post hoc* tests were applied to compare RTVs between groups. Statistical significance was set at *p* < 0.05.

## 3. Results

**Figure 2:**
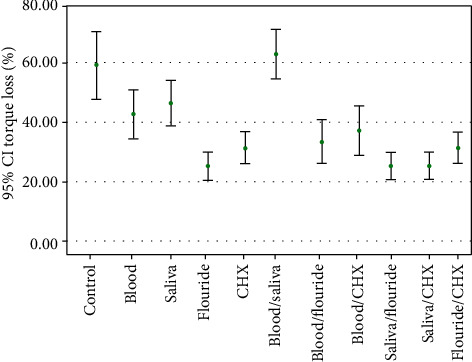
Comparison of the mean difference of reverse torque value in the studied groups.

Descriptive statistics and torque loss values of all groups are shown in Table 1. In addition, a comparison of the mean difference of RTV in the studied groups is represented in Table 2. The results indicated that RTV values were less than initial torque (30 Ncm) in both of the control and contamination groups. The highest RTV was observed in the fluoride group (26.00 Ncm, SD: 1.02). In other groups, this rate for the control, blood, saliva, and chlorhexidine groups were 18.00 ± 1.78 Ncm, 22.12 ± 1.56 Ncm, 21.56 ± 1.43 Ncm, and 21.89 ± 1.25 Ncm, respectively. In combination groups, the highest RTV was observed in the saliva+CHX group (23.89 ± 1.92 Ncm). In other combination groups, this rate for the blood+CHX, blood+saliva, saliva+fluoride, fluoride+CHX, and fluoride+blood groups were 22.56 ± 1.73 Ncm, 22.00 ± 1.54 Ncm, 20.11 ± 1.58 Ncm, 23.51 ± 1.119 Ncm, 21.02 ± 1.38 Ncm, and 20.11 ± 1.58 Ncm, respectively. In addition, the contamination groups' RTV means were more than the control group, and this difference was statistically significant for the blood (*p* = 0.04), chlorhexidine (*p* = 0.03), and fluoride groups (*p* = 0.01). Despite a higher RTV of the saliva group than the control group, it was not statistically significant (*p* = 0.21). Furthermore, despite higher RTV of the blood group, there was no statistically significant RTV mean difference between blood and saliva groups (*p* = 0.82). In addition, despite the higher RTV of the fluoride group, there was no statistically significant RTV mean difference between fluoride and chlorhexidine groups (*p* = 0.58). Of note, in comparison with combination groups, RTV mean was more than the control group and this difference was statistically significant for the saliva+CHX (*p* = 0.01), blood+CHX (*p* = 0.02), blood+saliva (*p* = 0.041), saliva+fluoride (*p* = 0.01), and fluoride+CHX groups (*p* =0.03) (Figure 2). A post hoc Tukey test showed that the control groups and contamination groups, except saliva, differed significantly at *p* < 0.05. This test also showed that control groups and combination groups differed significantly at *p* < 0.05. In addition, *post hoc* Tukey test showed that the blood group and fluoride group; saliva group and CHX group; and saliva group and fluoride group differed significantly at *p* < 0.05.

## 4. Discussion

This study is aimed at investigating whether the contamination of abutment screws with blood, saliva, blood, fluoride, and chlorhexidine or a combination of them would affect the initial torque value. Based on the results, RTV mean was less than the initial torque (30 Ncm) in both of the control and contamination groups. This result is in agreement with other studies that showed a lower torque is necessary for screw loosening relative to its initial torque [8, 19, 20]. In the current study, the maximum RTV mean was observed in the fluoride group (26.00 ± 1.02 Ncm). In other groups, this rate for the control, blood, saliva, and chlorhexidine groups were 18.00 Ncm (SD: 1.78), 22.12 Ncm (SD: 1.56), 21.56 Ncm (SD: 1.43), and 21.89 Ncm (SD: 1.25), respectively (Table 1). In combination groups, maximum RTV mean was observed in the saliva+CHX group (23.89 ± 1.92 Ncm). In other combination groups, this rate for the blood+CHX, blood+saliva, saliva+fluoride, fluoride+CHX, and fluoride+blood groups were 22.56 ± 1.73 Ncm, 22.00 Ncm ± 1.54, 20.11 ± 1.58 Ncm, 23.51 ± 1.19 Ncm, 21.02 ± 1.38 Ncm, and 20.11 ± 1.58 Ncm, respectively. Based on our results, the null hypothesis was rejected. These results are not similar to that of Haack et al. [10] and Kano et al. [21]. This difference may be due to the difference in the structural components of the implants which may be different in the various implant design. Depending on the implant design, the magnitude or type of forces applied to the bone-implant interface can be affected [10, 21]. In addition, the difference in RTV between studied groups may be due to the different features of contamination factors (CHX and fluoride). Our results can be supported by Kozlovsky et al. [22] who reported that the implant surface roughness could impact chlorhexidine adhesion to the implant. Furthermore, in our study, the fluoride group had less percentage of RTV which may be due to its lubricating feature. Moreover, the effect of saliva contamination in the implant hole on the RTV was also assessed in a previous study. Lower torque loss in the salivary group than the control group in this study was not statistically significant. This result is similar to the findings of Gumus et al. [18] but is in contrast to the results of Micarelli et al. [23] and Asli et al. [24]. They stated that saliva as a lubricant increased the RTV. In addition, chlorhexidine, compared to control, increased the RTV, which may be due to the lubricating feature of chlorhexidine. This result was similar to the obtained findings by Guda et al. [25] and Asli et al. [24]. They reported that chlorhexidine increases the RTV while Micarelli et al. [23] showed that chlorhexidine decreases the RTV. Also, contrary to these results, Gumus et al. [18] found that contamination with chlorhexidine has no statistically significant effect on RTV. Different methodology and variations in the form of contamination (for saliva; natural vs. artificial and for chlorhexidine; gel vs. liquid; wettability) and concentration may also account for differences in results between studies. In addition, the lack of simulating oral condition seems to be an effective factor for variation in reported results [18, 26, 27].

Despite the relatively high risk of blood and fluoride contamination of the abutment screw in clinical practice, a few studies on blood and fluoride contamination of the abutment screw have been reported in the literature. The findings of Gumus et al. [18] suggest that the accumulation of blood on the surface of the abutment screw, through the creation of a biofilm, can have a negative effect on RTV. Consistent with these results, we showed that blood contamination decreases the initial torque value. It has been found that the viscosity of blood may have an important role in the RTV reduction after contamination [18]. Compared to blood, the saliva has a lower viscosity (natural saliva has a viscosity of 1.9 cP, blood has a viscosity of 3.33 cP.); therefore, such a difference in viscosity could explain the differences between saliva and blood in terms of their effect on RTV [18, 28].

Although the authors consider the results of the current study to be valid, several certain limitations may be acknowledged. In our study, contaminations may occur at the implant-abutment connection after abutment torquing during prosthesis function; therefore, in further research, it is suggested to investigate the effect of contamination of implant-abutment specimens with different substances under cyclic loading conditions.

## 5. Conclusion

In conclusion, RTV values of all contaminated groups were less than initial torque and RTV for all groups from minimum to maximum were as follows: control group<saliva and blood groups<fluoride and chlorhexidine groups. In addition, in combination groups, maximum RTV mean was observed in the saliva+CHX group which was less than the initial torque. Further studies, considering in vivo conditions, are needed to prove the validity of our results. The obtained results provide clinicians with new insight into the effect of probable fluid contaminations in the implant-abutment interface.

## Figures and Tables

**Table 1:** Descriptive statistics and torque loss values of all groups.

	N	Mean	Std. deviation	Std. error	95% confidence interval for mean		Minimum	Maximum	Torque loss
					Lower bound	Upper bound			
Control	44	18.00	1.78	0.62	17.56	20.43	17.00	23.00	36.70%
Blood	44	22.12	1.56	0.51	19.93	22.29	19.00	23.00	29.7%
Saliva	44	21.56	1.43	0.44	19.53	21.58	19.00	22.00	31.70%
Flouride	44	24.00	1.02	0.37	23.14	24.85	23.00	26.00	20.00%
CHX	44	21.89	1.25	0.38	21.99	23.78	21.00	25.00	23.70%
Blood+saliva	44	22.00	1.54	0.42	19.16	21.53	18.00	25.00	29.70%
Blood+flouride	44	20.11	1.58	0.71	20.73	24.29	17.00	24.00	28.7%
Blood+CHX	44	22.56	1.73	0.54	21.53	22.18	19.00	23.00	30.80%
Saliva+flouride	44	23.51	1.19	0.47	23.51	23.15	21.00	25.00	28.00%
Saliva+CHX	44	23.89	1.92	0.68	20.89	24.58	22.00	26.00	29.60%
Flouride+CHX	44	21.02	1.35	0.58	21.19	22.48	20.00	25.00	28.10%

**Table 2:** Comparison of the mean difference of reverse torque value in the studied groups.

Tukey HSD					
Study groups		Mean difference	95% confidence interval		
			Lower bound	Upper bound	*p* value
Control	Blood	-2.11	-4.04	-0.18	0.041
Control	Saliva	-1.56	-3.48	0.37	0.16
Control	Fluoride	-5.00	-6.93	-3.07	0.033
Control	CHX	-3.89	-5.82	-1.96	0.011
Blood	Saliva	0.56	-1.37	2.48	0.92
Blood	Fluoride	-2.89	-4.82	-0.96	0.001
Blood	CHX	-1.78	-3.71	0.15	0.08
Saliva	Fluoride	-3.44	-5.37	-1.52	<0.001
Saliva	CHX	-2.33	-4.26	-0.41	0.01
Fluoride	CHX	1.11	-0.82	3.04	0.48
Blood+saliva	Control	-2.14	-7.11	5.89	0.04
Blood+fluoride	Control	-2.08	-3.96	-0.10	0.04
Blood+CHX	Control	-4.1	-2.30	0.48	0.021
Saliva+fluoride	Control	-3.5	-4.29	-0.38	0.01
Saliva+CHX	Control	-3.75	-7.9	-2.07	0.01
Fluoride+CHX	Control	-5.12	-6.95	-1.30	0.031

The mean difference is significant at the 0.05 level.

## Data Availability

All data generated or analyzed during this study are included in this article. Further enquiries can be directed to the corresponding author.
